# The Demographic Benefits of Belligerence and Bravery: Defeated Group Repopulation or Victorious Group Size Expansion?

**DOI:** 10.1371/journal.pone.0021437

**Published:** 2011-07-05

**Authors:** Laurent Lehmann

**Affiliations:** Department of Ecology and Evolution, University of Lausanne, Lausanne, Switzerland; University of Maribor, Slovenia

## Abstract

Intraspecific coalitional aggression between groups of individuals is a widespread trait in the animal world. It occurs in invertebrates and vertebrates, and is prevalent in humans. What are the conditions under which coalitional aggression evolves in natural populations? In this article, I develop a mathematical model delineating conditions where natural selection can favor the coevolution of belligerence and bravery between small-scale societies. Belligerence increases an actor's group probability of trying to conquer another group and bravery increase the actors's group probability of defeating an attacked group. The model takes into account two different types of demographic scenarios that may lead to the coevolution of belligerence and bravery. Under the first, the fitness benefits driving the coevolution of belligerence and bravery come through the repopulation of defeated groups by fission of victorious ones. Under the second demographic scenario, the fitness benefits come through a temporary increase in the local carrying capacity of victorious groups, after transfer of resources from defeated groups to victorious ones. The analysis of the model suggests that the selective pressures on belligerence and bravery are stronger when defeated groups can be repopulated by victorious ones. The analysis also suggests that, depending on the shape of the contest success function, costly bravery can evolve in groups of any size.

## Introduction

Coalitional aggression between groups of individuals occurs not only in humans but is prevalent in other vertebrates and invertebrates as well. For instance, colonies of army ants battle against each other to increase the size of their territory [1] and termites satisfy the condition of eusociality by the existence of sterile soldiers, not because they produce sterile workers [2]. Groups of chimpanzees engage in coordinated raids to obtain additional mates and territory [3], [4], and prehistoric groups of hunter-gatherers fought against each others for resources by throwing spears and boomerangs. It has even been suggested that a typical tribal society lost about 5 percent of its population in combat each year [5].

Coalitional aggression is a group level strategy, which allows individuals within groups to acquire reproduction enhancing resources from other groups. This strategy might have played a central role in human evolution [6]–[12]. While modern warfare between large scale societies is unlikely to be directly driven by motives of reproduction, it is important to try to understand the conditions under which coalitional aggression may have been selected for in natural populations [12], [13]. This strategy is individually costly in time and energy. What then are the rewards that make this enterprise worthwhile so that it can be selectively favored over an evolutionary time scale?

While the analytics of the proximate causes of raiding is well developed [14]–[18], there are few analyses of the demographic benefits leading to the evolution of intraspecific coalitional aggression between small-scale societies, and existing ones consider a saturated habitat and assume that the benefits of warfare come through the repopulation of defeated groups. In this case, individuals from a victorious group of a pairwise contest migrate into the defeated group and partially or completely replace individuals from the defeated group, thereby adopting its territory and/or its females [19]–[22]. This scenario may be consistent with a situation where victorious groups take over specific resource points that were occupied by defeated ones. Fights in social insects may occur over colonies lying in suitable habitats [1] and primitive warfare in hominids occurred over crucial water points and shelters [12], [23].

But repopulation of defeated groups is not the only plausible demographic benefit that may lead to the evolution of coalitional aggression. An alternative situation is that resources are captured from defeated groups by victorious ones, which, when used by the the individuals of victorious groups may result in an increase in their local carrying capacity. For instance, due to its high nutritional value, meat of all sorts was contested among hunter-gatherers [12], [23]. Groups winning contests over animal populations may have obtained higher shares of the contested resources. The local use of such resources may then lead to different demographic outcomes than repopulation of defeated groups. For instance, an increase in size of victorious groups may not only increase their contribution to the ancestry of the population because they are larger, but may also increase the probability that these groups win further battles. A self-enforcing demographic loop leading to higher levels of warfare between groups is thus a plausible outcome of this process.

The aim of this paper is to analyze the co-evolutionary dynamics of belligerence and bravery under this alternative demographic scenario, where victorious groups can expand in size as an outcome of warfare. These two traits are likely to be involved in coalitional aggression as belligerence increases an actor's group probability of trying to defeat another group and bravery increases an actors's group ability to defeat another group. In order to be able to contrast the selective pressure on belligerence and bravery under the two aforementioned demographic scenarios, I present a mathematical model, which takes both scenarios into account and allows me to contrast how different assumptions may lead to similar or different selective pressures on the evolution of these two traits when interaction occurs between small-scale, pre-state societies (“hunter-gatherer” warfare). The analysis of the model suggests that the selective pressure on belligerence and bravery are stronger when defeated groups can be repopulated by victorious ones.

## Analysis

### Life cycle assumptions

The population is assumed to consist of an infinite number of groups, which are connected by random dispersal (i.e., infinite island model of dispersal; [24]). The natural environment is assumed to allow only 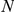 individuals to reach adulthood in each group. The life cycle of individuals living in this population is assumed to be similar in baseline structure to an earlier analyses of the evolution of coalitional aggression [22], but it is augmented with the possibility that the sizes of groups can vary as an outcome of warfare; namely, the groups can become larger or smaller than the baseline size 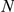 determined by the environmental condition. Because this feature adds more complexity to the model, individuals within groups are assumed, for simplicity, to be haploid and follow clonal reproduction. For ease of presentation of the demography underlying the model, I begin by introducing the basic demographic assumptions and model parameters by assuming a monomorphic population, where all individuals express the same phenotypes. I then introduce variation in behaviors and evolutionary dynamics.

**Figure 1:**
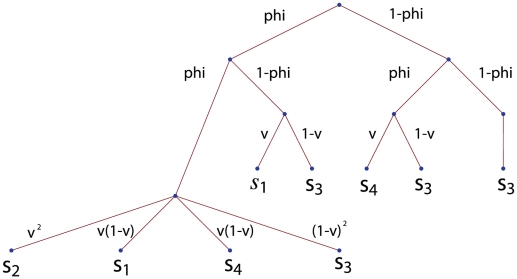
Demographic outcomes and events faced by the individuals in a focal group (the terms “outcomes” and “events” are used as defined in probability theory, e.g., p. 18, chapter 1 of [**62**]). Each different connected series of edges starting at the root node (top of the figure) provides a demographic outcome. Different outcomes may lead to similar demographic events, which are denoted by 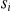 and described in the text. The first series of edges, directly issued from the root node, represents the probabilities that the focal group fights or does not fight to conquer another group from the population. The second series of edges represent the probabilities that the focal group fights or does not fights locally. The third series of edges represent the winning probabilities of the various battles, which occur only if there is a fight. For instance, the left most series of connected edges represent the outcome where the focal group fights against another group upon attacking (probability 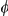), it fights locally because it is attacked (probability 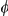) and wins the two battles (probability 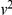). In order to obtain the probabilities of occurrence of each demographic event describes in the text, one has to sum the probabilities of occurrence of outcomes where that event obtains, which gives 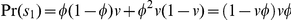, 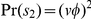, 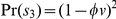, and 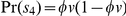.

In each generation, the following events occur in sequence. (1) Each adult individual in a group produces a large number of juveniles. Juveniles mature and become subadults. (2) Each subadult disperses with probability 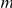 to a new randomly chosen group. (3) War occurs between groups. With probability 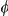 the subadults in each group engage into a fight with another group from the population and try to defeat it in order to gain its resources. The attacker group wins the ensuing battle with probability 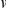. The individuals from each group might also engage into a fight locally, to defend their group because it is attacked. This also occurs with probability 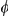, in which case the attacker wins the fight with probability 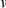. Each group is assumed to engage only in a single fight locally (to defend its resources), but a focal group can be both an attacker and a defender in the same generation (see Fig. 1). (4) Adults die and density-dependent competition (regulation) occurs in each group between subadult individuals to form the next generation. The number of individuals that reach adulthood in each group depends on the outcomes of warfare and two different but complementary types of demographic scenarios will be analyzed:

(i) Defeated group repopulation scenario (DGR). Here, in groups that have not been defeated, only subadults from that group compete against each other for access to breeding spots vacated by the death of adults. By contrast, in groups that have been defeated, subadults from both the victorious and defeated group compete against each other for vacated breeding spots. With probability 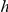 an individual randomly sampled from a defeated group after competition is assumed to be a member from that group before competition. Hence, defeated groups are partially repopulated by individuals from the victorious group and 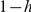 can be though of as the fraction of the individuals in a defeated group that are replaced by those of the victorious group. In both defeated and non-defeated groups exactly 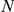 individuals are assumed to reach adulthood.

This DGR scenario is essentially a haploid version a the model mentioned above [22], but different assumptions will be employed below for the behavior of individuals in the population. Further, the interactions between hostile groups will be made more mechanistic here, which complements the previous analysis and will also clarify its results.

(ii) Victorious group size expansion scenario (VGE). Here, instead of repopulating a fraction 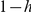 of defeated groups, individuals from the victorious group extract a share 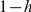 of the resources of defeated groups and use them in their natal group to produce offspring. Alternatively, this scenario could be interpreted as a situation where two groups contest for a given prize (e.g., some resources found in the environment) and where the winner of the contest obtains a fraction 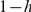 of the prize.

In order to be able to directly compare this VGE scenario to the DGR scenario described above, I assume that the extraction of resources from defeated groups leads to an increase in the number of individuals reaching adulthood in victorious groups by 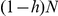 individuals, while only 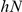 individuals survive to adulthood in defeated groups. The increase (or reduction) in group size is assumed to last only a single generation because it is reasonable to postulate that the additional resources gained through warfare can only sustain additional individuals over a single generation. Gained resources are thus temporary and there is no inheritance of resources across generations.

One may also suppose that warfare occurs at different timings in the life cycle of the individuals. For instance, subadults within groups may wage war before their dispersal instead of after dispersal so that stages (2) and (3) in the life cycle are interchanged. This case will also be investigated below.

### Demographic events

Because a focal group in a focal generation may either attack or not attack another group, fight or not fight a battle when it attacks another group, be attacked or not be attacked by another group, and win or loose any of the possible battles, several different outcomes may affect the fitness of an adult individual from a focal group (Fig. 1). It follows from the assumptions of scenario (i) and (ii) that the different outcomes can be gathered into four different demographic events (Fig. 1). The first, denoted 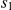, is when the focal group keeps all its resources and its individuals obtain a share of the “resources” of another group (be it through repopulation or by taking a share of the group's material resources). This event occurs when the focal group attacks another one, defeats the attacked group and is not defeated locally. The second demographic event, denoted 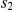, is when the focal group loses a share of it resources because it is defeated locally but obtains some resources from another group after attacking and winning the battle. The third event, denoted 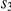, is when the focal group does not lose any resources locally and does not gain any resources from another group. Finally, the fourth event, denoted 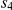, occurs when the focal group loose resources locally (as it is defeated) and does not obtain any resources from another group. This is the worst event for a group.

Under the DGR scenario, a demographic event determines the expected number of groups in which the individuals of a focal group may reproduce. For the events 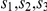, and 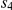, this number is, respectively, 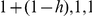, and 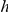 and thus varies between zero and one [for event 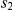, the mean of one is obtained as 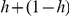]. For the VGE scenario, a demographic event determines the number of individuals that reach adulthood in the focal group. For the events 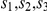, and 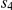, this number is, respectively, 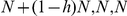, and 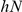 [for event 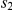, 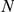 is obtained as 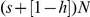]. Because groups are of size 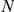 under the DGR scenario, the above assumptions imply that the additional number of descendants accruing to a group when it wins a battle is the same under the two scenarios and is given in both cases by 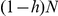. This increase in group level benefit only lasts a single generation, because, as assumed above, offspring do not inherit resources from the parental generation.

The demographic events experienced by each group may also change across generations. A focal group may experience a certain demographic event, say 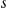, in a parental generation, and after one iteration of the life cycle that group may experience a different demographic event, say 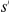, in the offspring generation. The changes in the demographic events between parental and offspring generation lead to a demographic dynamics, which is described by a stochastic process whose transition probabilities are affected by the behaviors of the individuals in the population.

### Evolving behaviors and selection gradient

Following earlier models for the evolution of warfare in small scale societies, individuals within groups are assumed to express two different traits [20], [22]. The first trait, denoted 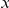, is called “belligerence”. The average level of belligerence in a focal group is assumed to vary between zero and one and it affects the probability 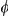 that a focal group fights another one. The second trait, denoted 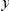, is called “bravery” and it is assumed to increase the probability 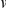 that a group fighting another one wins the battle. Bravery should be understood metaphorically as it may also represent the production of a sterile soldier caste in social insects or, for hominids, the investment into some technology of appropriation.

Although both belligerence and bravery increase group success, both traits are assumed to reduce the probability that an individual expressing them survives up to the stage of density-dependent competition (stage 4 of the life cycle). Since clonal reproduction is assumed, the average phenotype of an offspring is the same as that of its parent. One can then express the relative number of juveniles, which reach the stage where they compete for breeding spots, and that are produced by a focal adult individual with level of belligerence 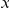 and bravery 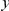 as 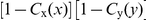, where 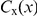 is the cost of expressing belligerence and 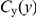 the cost of expressing bravery (both cost functions are as assumed to be convex). By contrast to previous work, this formulation entails that belligerence and bravery are costly even if the group of the actor is not engaged into a contest. There are two reasons for this assumption. First, the model is simpler to analyze. Second, the model may also represent battles between colonies of social insects, in which case the production of a soldier caste is made independently of whether there is a battle between groups or not. More generally, one may also assume that investment into belligerence and bravery may be costly to the actor throughout its life because these traits are probably costly in time and energy beyond the battlefield (e.g., belligerence may involve spending time convincing group members of raiding other groups and bravery developing the technologies of appropriation).

The evolutionary dynamics of belligerence (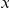) and bravery (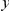) are complicated to analyze in full form because the model is demographically explicit (groups may vary in size), evolution occurs in a subdivided population (fluctuations of gene frequencies between groups occurs), and individuals within groups are subject to a varying environment (warfare is a stochastic process, which further depends on the behavior of individuals in the population). I thus employ the assumptions of weak selection and additive gene action. With these assumptions, one can evaluate the evolutionary dynamics of a focal trait through a phenotypic gradient approach by holding the other trait constant. Each trait is then assumed to follow a gradual, step-by-step transformation caused by the successive invasion of mutant alleles having different phenotypic effects than resident alleles fixed in the population (e.g., adaptive dynamics approach or ESS method; [25]–[30]).

The change in the frequency 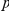 in the population of a given mutant allele, which codes for a small phenotypic deviation 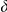 relative to the phenotype 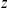 expressed by individuals carrying a resident allele (where 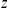 stems either for belligerence, 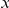, or bravery, 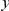), can be written for the infinite island model as 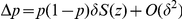, where 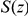 is the force of directional selection on the mutant allele and 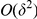 is a remainder that includes higher order terms [29]. Importantly, the selection gradient 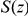 on the trait is independent of the frequency of the mutant in the population, which allows one to evaluate a candidate evolutionary stable strategy 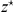 (ES level of belligerence or bravery) by solving 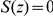 for 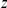. Evaluating the criteria of continuous stability [26], [28], [29] of the phenotype 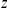 requires one to evaluate the change of gene frequency 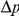 to the second order in 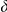 [29], [31], which is theoretically possible but a difficult task under the present demographic assumptions. However, the aim of this paper is to compare the forces of directional selection on belligerence and bravery under the VGE and DGR scenarios, which can be carried out by studying the gradient 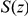 since higher order terms will be less important.

Although such an ESS approach does thus not give a full picture of evolution, it allows one to obtain explicit analytical approximations, which would otherwise be out of reach, and that have repeatedly shown to provide good predictions for long-term phenotypic evolution and/or the direction of selection under a wide spectrum of biological applications involving local genetic drift under complex demographies with weak assumptions about the distribution of mutant's deviating phenotypic effects [28], [32]–[37].

For the DGR scenario, group size is the same in each group in the population in each generation. There are also no correlations between the demographic events experienced by a group from one generation to the next, and the occurrence of warfare is independently distributed across generations. The selection gradient on the trait 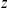 can then be evaluated by a standard application of the direct fitness method [29], [38], [39], as
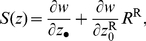
(1)where 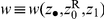 is the fitness function giving the expected number of adult offspring of a focal adult individual (an individual taken at stage 1 of the life-cycle) bearing a mutant allele and 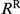 is the probability of identity-by-descent between the focal individual and a randomly sampled individual (including himself) from the focal patch, which is a measure of relatedness between group members. The derivatives of 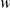 are the effects of actors on the fitness of a focal individual and are evaluated at the phenotypic value of the resident allele (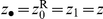), where the actors are the focal individual itself with phenotype denoted 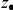, individuals from the focal group with average phenotype denoted 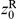 (the average is over all individuals from the focal group, thus including the focal individual itself), and individuals from different groups with average phenotype 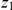.

Owing to the infinite island model assumptions, individuals from different groups (with phenotype 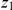) have zero relatedness to the focal individual. One way of understanding this result is by noting that when there is an infinite number of groups, the ancestral lineages of two homologous genes sampled from two individuals within the same group have either stayed in the same group and coalesced, or have migrated to two different groups and can then be considered as being independent [40], [41]. The relatedness coefficient 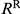 measures the increase in the probability of identity between genes sampled from individuals within the same group due to coalescence events within groups (e.g., “identity-by-descent”). By contrast, the ancestral lineages of two genes sampled from individuals from two different groups do not coalesce in a recent past and can be considered as being independent [40], [41]. Hence, these individuals are unrelated as their homologous genes are not more identical than two genes sampled at random from the population.

For the VGE scenario, group size may fluctuate across generations and correlations between demographic states may develop over time. The selection gradient 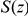 will then also depend on the reproductive values of individuals facing different demographic events, which take into account the differences in expected future contribution to the population of these individuals according to the different types of demographic events that occur in the groups they settle in [34], [42]. As a result, the form of the selection gradient 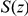 for the VGE scenario is more complicated than eq. 1 and is presented in Appendix S2 (eqs. B-1–B-2), but conceptually it is an extension of eq. 1, which includes demographic classes and where marginal fitness components are weighted by reproductive values.

### Fitness components for the DGR scenario

I will present the derivations of the fitness function 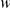 and the selection gradient 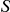 for the DGR scenario directly in the main text and refer to Appendix S2 for the derivation of these quantities for the VGE scenario, as it is more complicated in the latter case but conceptually similar.

In order to evaluate 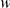 for the DGR scenario, or more generally the various components of the fitness function for the VGE scenario, we need to relate behaviors to fighting (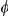) and winning (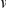) probabilities. These will determine the demographic events, which, in turn, determine fitness. The fighting and winning probabilities can be thought of as macro demographic variables, which depend on the mechanistic details relating trait value expression by individuals within groups to group level behavior.

#### Fighting probability

Owing to the infinite island model of dispersal assumptions introduced above, I assume that the probability that the focal group with subadults expressing average level of belligerence 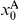 enters into a fight with another group is given by some function 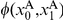, which also depends on the average level 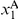 of belligerence among subadults in other groups in the population. Because any class of individuals from other groups (immigrants, juveniles, or adults) have zero relatedness to the focal individual, their average phenotypes can be considered to be equivalent and we can set 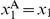. Because warfare occurs after the dispersal of juveniles (or subadults), the average phenotype among subadults in the focal group is related to that of adults before dispersal by
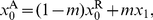
(2)since after dispersal a fraction 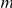 of offspring in the focal group are descendant from that group (with parents carrying average phenotype 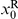), while a fraction 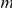 of offspring have immigrated from other groups (with parents carrying average phenotype 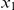).

Alternatively to the situation described by eq. 2, one may also assume that warfare occurs right before the dispersal of juveniles, in which case the average phenotype among subadults is the same as that among adults so that 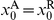. These two different timings of warfare can be considered simultaneously by writing the average phenotype among subadults as
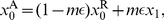
(3)where the parameter 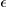 is equal to zero when warfare occurs before the dispersal of offspring and equal to one if it occurs after the dispersal of offspring.

The exact functional relationship relating 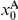 and 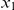 to the fighting probability does not affect the qualitative result reported below, but in order to obtain quantitative results one needs such a functional relationship. For this reason, I introduce the island model of warfare. Here, a group with average level of belligerence 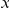 attacks independently another random group from the population with probability 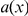, which is assumed to be an increasing function of its argument. Namely, the focal group attacks another group with probability 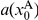, while another group from the population attacks a group sampled at random with probability 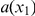. The assumption that each group attacks independently of each other another random group from the population implies that several groups may simultaneously attack the same group. Since resources are limited, I assume that during a single generation a focal group engages only into a single pairwise contest over its local resources with another attacker group. When several groups simultaneously attack the focal group (synchronously or asynchronously), one randomly sampled group among the attackers is assumed to engage into a fight with the focal group.

From these assumptions, the probability that the focal group engages into a fight locally is given by 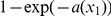, which increases with the level of belligerence 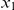, while the probability that the focal group enters into a fight with another group from the population through attacking is
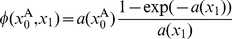
(4)(eqs. A-1–A-5 of Appendix S1). This equation says that the fighting probability of the focal group increases with the average level of belligerence 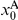 of its members, but decreases with the average level of belligerence in other groups in the population because, the higher the level of belligerence in the population, the lower the probability 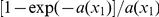 that the focal groups enters into a fight with a given group conditional on it attacking. Note that in a monomorphic population, for instance when 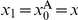, the fighting probability 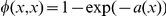 increases with the overall level of belligerence.

#### Winning probability

Eq. 4 gives the probability that a focal group engages in a fight. But we also need an expression for the probability that it wins the ensuing battle. I assume that the probability that the focal group, where subadults express average level 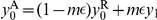 of bravery, and that attacks another group, where subadults express average level of bravery 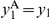, wins the ensuing battle is given by
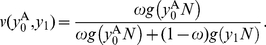
(5)


In this winning probability, 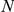 represents the number of individuals producing subadults, 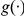 describes the power of contestants, which is a positive increasing function of its argument, which can be thought of as the total effort put by a group into the battle (level of bravery times relative number of combatants), and the parameter 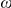 allows one to tune the advantage of being offensive. If 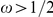, there is advantage to attacking if everything else among the two groups is held constant (e.g., 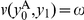 in a monomorphic population, where 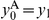). An offensive advantage may be justified by the fact that it has been suggested that most primitive warfare consisted of ambushes and ambuscades, which result from unilateral actions conducted under conditions where individuals from attacked group were caught helpless [12], [43]. On the other hand, a defensive advantage (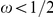) has been justified by the fact that attackers often don't know the intruded area as well as defenders, which puts attackers at a disadvantage [44].

Equation 5 is a so-called contest success function [14], [45], [46], which depends on the ratio of the effort the two opposing parties put into winning a battle. Such a functional relationship follows from a series of assumptions about contests [46], the strongest of which is probably that the contest among a smaller number of combatants is qualitatively similar to those among a large number of combatants.

#### Probabilities of occurrence of demographic events

In order to evaluate 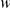 we need the probabilities of occurrences of the four demographic events. Let us use the shorthand notations 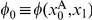 and 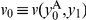, where the second expression is the probability that the subadults in the focal group win the battle upon entering into a fight with another group from the population. Likewise, call 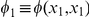 the probability that the subadults from another group in the population try to conquer the focal group and enter into a fight with it, and let 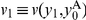 be the probability that the attacking group wins the ensuing battle. With these notations and using the different outcomes in Fig. 1, the probabilities that the events 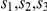, and 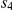 obtain in the focal group are, respectively, given by
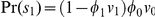


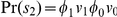


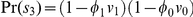


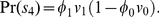
(6)


#### Fitness function

It is convenient to write the fitness function as
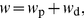
(7)where 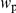 is the expected number of the focal individual's settled offspring that remained philopatric and 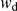 is the expected number of the focal individual's settled offspring in other groups after dispersal. Let us further call 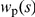 the expected number of philopatric offspring of a focal individual breeding in a group when demographic event 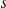 occurs. Averaging over all demographic events given in eq. 6, the philopatric component of fitness in eq. 7 becomes
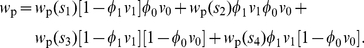
(8)


The fitness functions corresponding to each demographic event are evaluated by following the life cycle assumptions presented in the main text and standard calculations for the infinite island model [29], [41], [42], [47], which are as follows. A focal individual produces a relative number 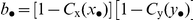 of offspring (relative to that of an individual in a resident monomorphic population) that reach the regulation stage (stage 4 of the life cycle of the main text). When the focal group has not been conquered, the focal individual's offspring, which remained philopatric (fraction 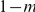 of 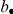), compete in the focal group with 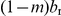 subadults produced in the focal group, where 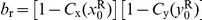 is the average relative number of subadults produced in the focal group that reach the regulation stage. The focal individual's philopatric offspring also compete against 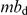 immigrants, where 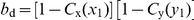 is the average relative number of subadults produced in other groups that reach the regulation stage.

When the focal group has been conquered, 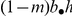 of the focal individual's offspring compete against 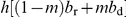 subadults reaching the regulation stage in the focal group after dispersal and warfare. The focal individual's offspring also compete against 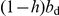 subadults from the victorious group. When the focal group conquers another group, 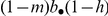 of the focal individual's offspring compete against 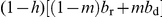 subadults from the focal group and 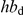 subadults from the conquered group.

If warfare occurs before the dispersal of subadults; that is, stages (2) and (3) of the life cycle are interchanged but every other assumption of the model is the same, the above fecundity functions still apply. These fecundity functions can be applied to both warfare occurring before or after the dispersal of subadults, because in both cases regulation occurs after dispersal and warfare and it is the number of offspring reaching the regulation stage that determines fitness. Hence, we can use the same fitness functions to analyze the two different timings of warfare by making use of eq. 3 and the analogous equation for bravery (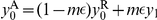).

Gathering all the above terms and taking into account the fact that, when the focal group conquers another group, the focal individual has descendants in the focal group and in the conquered group gives
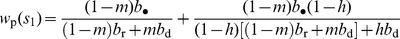


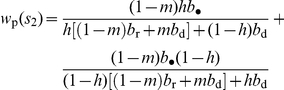


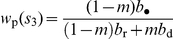


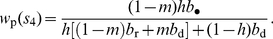
(9)


In order to evaluate 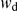, we need the probabilities that a random group from the population is in demographic states 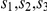, and 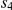. These probabilities are, respectively, 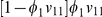, 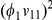, 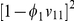, and 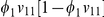, where 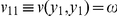 because the demographic events that obtain in other groups than the focal group depend only on the average level of belligerence and bravery in the population (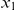 and 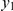). Averaging over all these cases, the immigrant component of fitness is
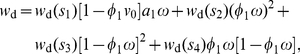
(10)where
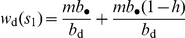


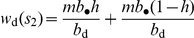


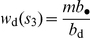


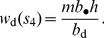
(11)


Substituting eqs. 9–11 into eq. 7 and simplifying yields the fitness of a focal adult as
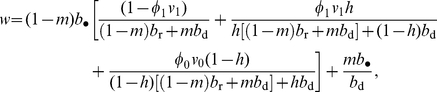
(12)where the philopatric component of fitness depends on three terms. The first term accounts for the fitness accruing to the focal individual when the focal group is not attacked plus when the group is attacked and wins the battle, the second term accounts for the fitness to the focal individuals when the focal group is attacked but loses the battle, and, finally, the third term accounts for the fitness to the focal individuals when the focal group attacks another group and wins the battle.

In order to compare the demographic benefits that accrue to a focal group under the DGR scenario to that obtained under the VGE scenario, it is useful to note that the reproductive value 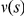 of a group in demographic state 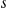, which is the asymptotic contribution of a group in state 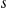 to the growth rate of the population (see eqs. B-5–B-6 of Appendix S2 and [34]) is obtained in the DGR scenario as 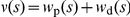, where the fitness functions are evaluated at the phenotypic value of the resident (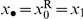 and 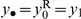), which gives
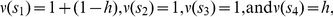
(13)and where the average of these group reproductive values over the demographic events is equal to one.

#### Probability of identity-by-descent

Having an explicit expression for the fitness function 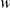 to substitute into eq. 1, it remains to evaluate the average probability of identity-by-descent 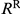 between two individuals sampled with replacement from the same group, which is carried out by using standard methods [29], [47]–[49] applied to the island model with warfare [50]. We have
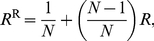
(14)where 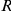 is the probability of identity between two individuals sampled without replacement from the same group and that satisfies the recursion
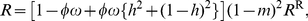
(15)where 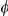 and 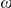 are, respectively, the fighting and winning probabilities in a population monomorphic for belligerence and bravery. Eq. 15 can be understood as follows. With probability 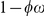 the focal group where the two individuals are sampled has not been defeated, in which case the probability of identity between the two individuals is 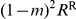: with probability 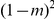 the two offspring descend from the same group, in which case they descend from the same individual with probability 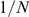 and then carry identical genes or they descend from two different individuals with probability 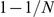 and then carry identical genes with probability 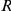. With probability 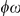 the focal group has been defeated by another group in which case the two individuals descend from the same group before regulation with probability 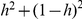 (with probability 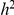 from the defeated group and with probability 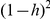 from the victorious group), and their probability of identity is then equal to 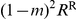 [50].

After simplification, eq. 15 reduces to
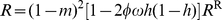
(16)and on substitution of eq. 14 and solving the for 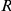 gives
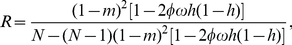
(17)which further yields
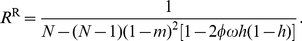
(18)


## Results

### Defeated group repopulation

#### Belligerence

Substituting the direct fitness function (eq. 12) and the probability of identity-by-descent (eq. 18) into eq. 1, using belligerence as the focal trait (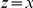), eq. 3 to describe the phenotype of subadults, holding bravery constant (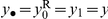), and applying the chain rule at the neutrality point (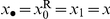), the selection gradient on belligerence can be expressed as
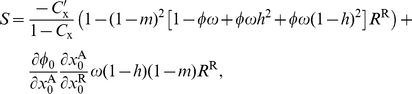
(19)where 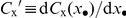. Using 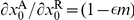, one has
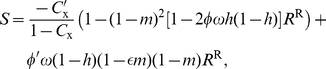
(20)where 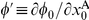 is the derivative of 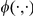 with respect to its first argument, which, when using eq. 4, becomes 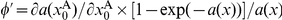.

Because 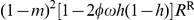 is equal to 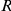 (eq. 15), the selection gradient reduces to
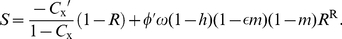
(21)Setting 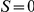, the candidate ES level of belligerence then satisfies the equation
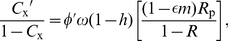
(22)where 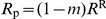 is the average probability of identity between a gene sampled in a philopatric individual and a homologous gene sampled from a neighbour [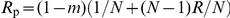], which can also be interpreted as the probability of identity between a gene sampled in a focal individual and one in an individual sampled at random from the same group in the parental generation.

The left member of eq. 22 is the marginal decrease 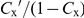 in fitness stemming from a single individual increasing its level of belligerence as a result of expressing a mutant allele relative to that of expressing a resident allele. The term 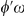 in the right member of eq. 22 is the marginal increase in the probability of conquering another group (change in contest probability times winning probability), which results from an individual expressing belligerence, and it tunes the fitness benefit of defeating an attacked group.

The increase in fitness stemming from defeating a group is given by 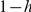 in eq. 22, which can be thought of as the average increase in the reproductive value of a victorious group, and is weighted by a measure of relatedness 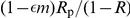 between group members, which depends on the model's parameters and functions (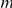, 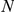, 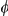, 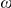, and 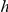). The term 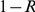 in the denominator reflects the reduction in the genetic variance in the population because interactions occur in a spatially structured population and 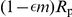 reflects the increase in the variance between groups. Eq. 22 shows that the more individuals are related within groups, the higher the level of belligerence.

When the right-hand-side of eq. 22 is higher than the left-hand-side, a higher level of belligerence is selected for, because the relatedness times the fitness benefits exceeds the fitness costs. When the right-hand-side of eq. 22 is lower than the left-hand-side, a lower level of belligerence is selected for. At an equilibrium point both sides of eq. 22 balance each other out. Substituting the explicit value of 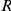 (eq. 17) into eq. 22, this equilibrium is given by
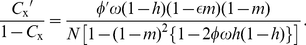
(23)When migration is low, the right member is large provided provided 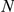 is not too big, in which case the selection pressure on costly belligerence is high. When migration increases, the right member decreases, which decrease the selection pressure on costly belligerence.

A special case of eq. 23 will be useful for comparing different results. This is the weak warfare benefit limit; namely, when 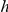 becomes close to one but nevertheless remains smaller than one (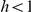), in which case eq. 23 reduces to
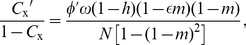
(24)where terms of order 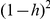 and of higher order in the right member of eq. 23 have been neglected.

#### Bravery

Substituting eq. 12 and eq. 18 into eq. 1, using bravery as the focal trait (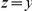), holding belligerence constant (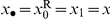), and following similar calculations as above, the selection gradient on bravery can be written as
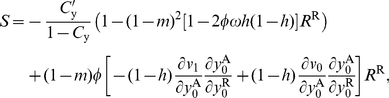
(25)where 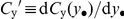, 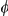 is the value of the fighting probability in a population monomorphic for the resident trait value. Using 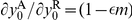, 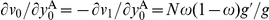, where 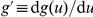, and defining
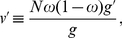
(26)which is the derivative of eq. 5 with respect to its first argument, the selection gradient can be further simplified to
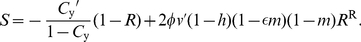
(27)


At an evolutionary rest point 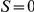, the candidate ES level of bravery then satisfies
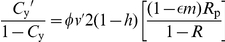


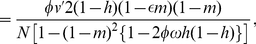
(28)which is similar in form to eq. 22. The left member of eq. 28 is the marginal decrease 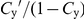 in fitness and 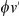 is the increase in the probability of conquering another group, which both stem from an individual expressing a mutant bravery allele. The benefit in the right member of eq. 28 takes the same interpretation as that in eq. 23 but is twice as large because bravery also increases the probability that the focal group wins a battle when attacked by another one and is thus not taken over.

#### Bravery in groups of very large size

The marginal change in the wining probability given by eq. 26 has the interesting feature of increasing with group size, 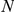, which suggests that, depending on the shape of the function 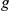 describing the power of contestants, the ES condition of bravery may become independent of groups size (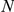 cancels out in the right member of eq. 28). Individually costly bravery may then evolve under very large group size. In order to see when this might be the case, it is useful to note that there are at least two meaningful ways to relate effort committed into fighting to the power function 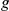 [14], [45], [46]. The first is to assume that the winning probability predicts contest outcome from the ratio of each side's effort 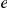 committed into fighting, in which case one can use 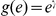, where 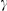 scales the decisiveness of fighting effort disparities [14], [45]. The second is to assume that the winning probability predicts the outcome of a battle from the difference of each side's effort committed into fighting, a situation that is described by the function 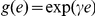, which leads to the contest success function being logistic. This difference form of the contest success function characterizes a situation where the outcome of a battle depends critically on just a little difference between the opponents force [45].

Using the ratio form of the contest success function gives 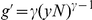 so that 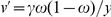, in which case the selection pressure on bravery decreases with group size and vanishes when groups become very large. For the difference form of the contest success function, one has 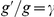 so that 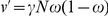, in which case the selection pressure on bravery becomes independent of 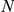 as this parameter cancels out in eq. 28. It follows that bravery may now evolve in groups of any size.

#### Relation to previous analytical models

An analytical model of selective groups extinction was developed in [19] (see also [51]), where altruists within groups increase the probability that a group wins a battle during pairwise contests. In this previous model, a parameter 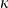 is used, which is defined as the likelihood that groups engage in a contest and is equivalent to the fighting probability, 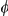, whereby we can set 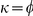. [19] also introduces a parameter 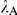, which is the change in a group's survival probability due to its members expressing altruism should a contest occur, so that one can write 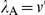. The cost 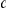 in [19] is also equivalent to 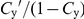 here and since complete repopulation of defeated groups was assumed in [19], one can also set 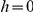. With all these stipulations, we can write eq. 28 as 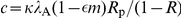, which agrees with eq. 6 of [19] in the absence of reproductive leveling (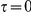) and if one makes the additional assumption that warfare occurs after dispersal, in which case 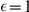 and 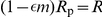 in eq. 28 if 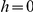. The result of [19] thus agrees qualitatively with those derived above.

Eq. 28 is also related to eq. 3.3 of [22], where the cost 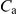 is given here by 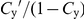, while the change 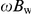 in winning probability is given here by 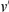. With this, we can write eq. 28 as 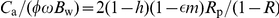. The right hand side of eq. 3.3 of [22] can be simplified to 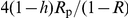 [50], so that this eq. 3.3 reads 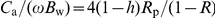. The difference in the factor 2 between the two results stems from the fact that in our earlier analysis we assumed a two-sex rather than a one-sex model and assumed that only males expressed belligerence, while both sexes benefited from it so that the cost of expressing belligerence is halved relative to the present model.

In addition, the term 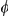 appearing in the denominator of the left-hand side of eq. 28 is missing in 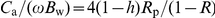 because we assumed previously [22] that bravery was costly only conditional on there being a contest between groups, which cancels the 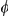 in the denominator of eq. 28. Finally, the absence of the term 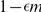 in eq. 3.3 of [22] shows that our earlier model involves warfare occurring before the dispersal of juveniles (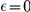), although we described in our life cycle section that warfare occurred after the dispersal of juveniles. Hence, in order to match the life cycle description, the benefit 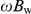 must be taken as being of order 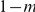 and the same reasoning applies to the invasion condition of belligerence [22, eq. 3.1]. Alternatively, our earlier model can be interpreted as a model of warfare before the dispersal of offspring.

These considerations show that DGR demographic scenario analyzed here is consistent with previous work endorsing closely related assumptions. The main difference with previous work so far is that the qualitative results obtained earlier apply to warfare occurring before and after the dispersal of subadults (the warriors in the model) and the winning and fighting probabilities have been made more mechanistic (e.g., eq. 4 and eq. 5). Further, making these functions more mechanistic has revealed that, depending on the form of the contest success function, costly bravery may actually evolve in large-scale societies and that the fighting probabilities are likely to depend on the level of belligerence in other groups from the population, which was not clear from previous work.

### Victorious group size expansion

Under the VGE scenario, the selection gradients on belligerence and bravery involves more terms because the reproductive value of offspring settling in groups facing different demographic events must be taken into account (compare eq. 1 to eqs. B-1–B-2 of Appendix S2). In addition, the stochastic process determining the probabilities of occurrences of the demographic events (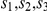, and 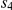) of a focal group depends on its size and the sizes of other groups because the contest success functions depend on the sizes of pairs of opposing groups in that case (eqs. B-14–B-15 of Appendix S2). With all these complications, I was unable to obtain explicit analytical expressions for the selection gradients on belligerence and bravery for all parameter values.

Nevertheless, it was observed in numerical work that the stationary probabilities of occurrences of the demographic states often take values very close to that of independently distributed probabilities of occurrences across generations, which are the stationary values of the demographic process when 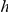 is equal to one. Hence, an approximation for this model is to consider that 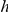 takes values close to one (a defeated groups keeps a large fraction of its resources), in which case one can obtain analytical expressions for the selection gradients on belligerence and bravery for the VGE model, which are now detailed.

#### Belligerence

Assuming that the fitness benefits of defeating other groups are small, we can evaluate the selection strengths on belligerence to the first order in 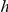 (i.e., neglecting terms of order 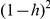 and higher order; eqs. B-23–B-32 of Appendix S2). The candidate ES level of belligerence is then found to satisfy
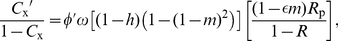
(29)where the left member has the same form as that in eq. 22. The term in square brackets in the right member of eq. 29 measures the average increase in a group's reproductive value from defeating another group, where the average is over the cases where the focal group is and is not defeated locally by another group. The second term in the right member of eq. 29 is a measure of relatedness, which has the same interpretation as that in the DGR scenario (eq. 22) and which is given by
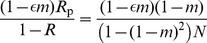
(30)(eq. B-38 of Appendix S2), whereby eq. 29 reduces to
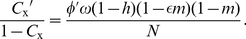
(31)


Comparing eq. 31 to eq. 24 highlights that the demographics benefits of belligerence are generally lower under the VGE scenario than under the DGR scenario and that this difference decreases when the level of migration, 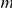 increases. The selection gradients on belligerence for the two demographic situations become equivalent when migration becomes very strong (set 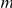 close to one in eq. 24 and eq. 31). The difference between the two selection gradients for small 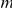 values stems from the fact that under the DGR model the benefits of obtaining additional resources from other groups are partially cancelled out as this also increases local competition. Indeed, an increase in the carrying capacity of victorious groups implies that there are more individuals competing to gain access to adulthood in such groups in the next generation. This is why the benefit of conquering other groups in eq. 29, 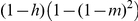, decreases as the level of philopatry increases.

**Figure 2:**
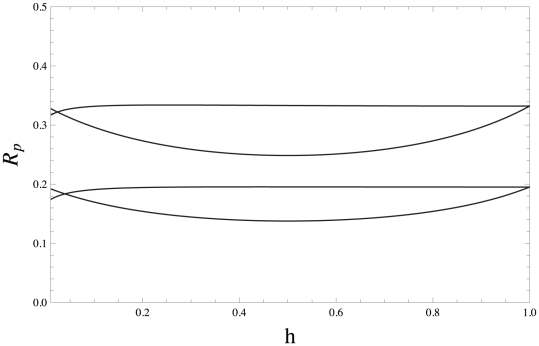
Graph comparing the probability of identity 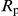 in the DGR scenario to that obtained in the VGE scenario. The quasi flat line is the the value of 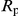 for the VGE scenario obtained without making any assumption on the value 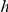 can take, while the downward bent line is the relatedness for the DGR scenario. The first set of two lines in the graph is for 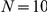, while the second set of two lines is for 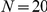, while the other parameter values are 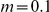, 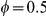, 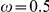, and 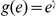, where the value 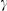 takes does not affect 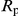.

The probability of identity 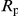 is higher under the VGE scenario than under the DGR scenario for most of the range of parameter values (Fig. 2). This stems from the fact that under the VGE model, defeated groups are repopulated by individuals from two groups, which increases the diversity within groups unless 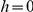 in which case groups are completely repopulated so that the relatedness within groups is not affected by the repopulation event. But by comparing eq. 31 to eq. 24, we see that the selective pressure on belligerence under the DGR scenarios is generally larger than that under the VGE scenario.

#### Bravery

Assuming that the benefits of defeating other groups are small (e.g., 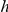 is close to one), the candidate ES level of bravery is found to satisfy
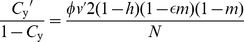
(32)(eq. B-43 of Appendix S2). Eq. 32 is similar in form to the selection gradient on bravery under the DGR model (eq. 22). But here again, comparing these two equations shows that the demographic benefits of bravery are generally lower under the VGE scenario than under the DGR scenario, which is again due to the fact that the benefits of obtaining resources from other groups are partially cancelled out as they also increase local competition.

#### Stronger warfare benefits

The analytical results presented in the last two section (eq. 31 and eq. 32) suggest that the selective pressure on belligerence and bravery is generally stronger under the DGR than under the VGE scenario, especially when migration is limited. However, these analytical results rely on the assumption that the benefits of warfare are small and one may wonder if these results still hold if the benefits of warfare are strong; that is, if 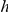 is low.

**Figure 3:**
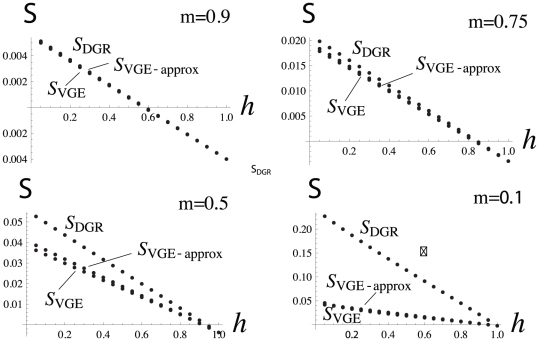
Selection gradient 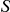 on belligerence for the DGR and VGE scenarios as a function of 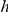. The functional relationships used are 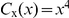, 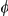 given by eq. 4 with 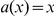, and 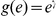, and the resident trait value was set to 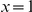. The parameter values are 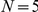, 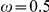, and the value 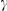 takes does not affect the selection pressure on belligerence. Further, 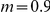 in the top right panel, 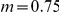 in the top left panel, 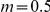 in the lower right panel, and 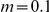 in the lower left panel. The top line in each panel is eq. 21; that is, the selection gradient for the DGR scenario and denoted by 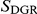. The second line in each panel is 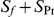 with eq. B-18 of Appendix S2 using 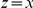 and eq. B-20 of Appendix S2; the selection gradient for the VGE without making any assumption on the value 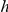 can take, which is denoted by 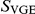. The last line in each panel is eq. B-36 of Appendix S2; the selection gradient for the VGE assuming that 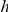 is small and is denoted by 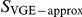. Three observations follow from this figure, which were also observed under a wider range of numerical exploration. First, for strong migration rates all three selective pressures agree. Second, for low migration rates the selective pressure is stronger under the DGR than under the VGE scenario for all values of 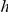. Third, the small 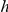 approximation of the VGE scenario underestimates the strength of selection on belligerence when migration is low, but the approximation works quiet will for 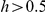. Similar results were observed if the contest success function is of the difference form.

Fig. 3 compares the selective pressure on belligerence under the VGE scenario to that obtained under the DGR scenario without making any assumption on the values 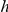 can take. Fig. 3 illustrates that the selective pressure on belligerence is always larger under the DGR than under the VGE scenario for all values of 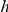. This suggests that the qualitative results obtained by assuming large 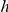 values hold more generally, and that the weak warfare benefit approximation (large 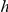 values) is actually often good for values as high as 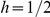, and the same results apply to the evolution of bravery. Similar patterns were observed for the selection pressure on bravery and using both the difference and the ratio forms of the contest success function.

## Discussion

Evolutionary models of social interactions occurring within and between groups of individuals have often emphasized the benefits and conflicts associated with resource exchange within groups [52]–[56]. But since resources come in total finite supply in a population, struggle and conflict between groups is also likely to be an evolutionary outcome [4], [6], [7], [10], [16], [19]. Here, the evolutionary dynamics of belligerence and bravery in the island model of warfare has been analyzed under two distinct but complementary types of demographic benefits that can drive group conflict: defeated group repopulation and victorious group size expansion.

The analysis of the models shows that the condition under which both belligerence and bravery spread in the population under the two demographic scenarios depends on four quantities (see eq. 22, eq. 28, eq. 29, and eq. 32). The first quantity is the relative marginal cost to a focal individual of expressing the trait under scrutiny [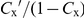 for bravery and 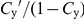 for belligerence]. The second is the increase in the probability of defeating another group from the population (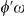 for belligerence and 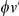 for bravery). The third quantity is the increase in the reproductive value of a group, which defeats another one, relative to that of not defeating it (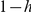 for the DGR scenario and 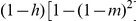 for the VGE scenario). This quantifies the demographic benefits of belligerence and bravery, which will affect the selection gradients on both traits proportionally to the relatedness between group members. The higher the relatedness within groups, the higher the reproductive value of a group belongs to the gene lineage carried by a focal individual expressing belligerence or bravery. The fourth quantity is thus a measure of relatedness between group members [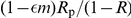]. Kinship, therefore, not only plays its classical effect in determining the individual's sacrifice in the promotion of its group [6], [53], [55], [56], but it also markedly affects the tendency of individuals within groups to try to take over other ones.

The increase in the probability of defeating other groups from the population (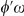 and 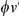) depends on the mechanistic details underlying the interactions between groups (e.g., contest success functions, how attack rates convert into fighting probabilities). Different ecological and environmental situations may result in different functional relationships mapping belligerence and bravery into fighting and winning probabilities. The strength of selection on bravery turns out to critically depend on such mechanistic details because effort committed into fighting may translate into very different wining probabilities [14], [45], [46]. For the case where contest outcome depends on the ratio of each side's fighting effort, the selection pressure on bravery decreases with group size and vanishes in large groups. By contrast, when contest outcome depends on the difference of each side's fighting effort, which may be a common situation [14], the selection pressure on bravery turns out to be independent of group size. This is interesting because it allows for the evolution of costly bravery in large-scale societies, without the need of invoking any special forces for that case.

How different are the selection on belligerence and bravery under the two demographic scenarios? It is worthwhile to emphasize at this point that in order to be initially able to compare the DGR to the VGE scenario, I assumed that the demographic benefits of warfare accruing to a focal group are comparable in magnitude. Although the demographic processes underlying these two models are different, the reproductive value 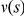 of a group in demographic state 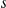, which provides the far distant future contribution of a group in state 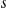 to the growth rate of the population (see eq. B-5 of Appendix S2 and [34], [57]), are exactly the same for the two scenarios when the migration rate of juveniles is complete and the distribution of demographic states is independent across generations [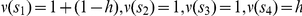, eq. 13 and eq. B-12 of Appendix S2]. In this case, the long-term fitness benefits accruing to an individual in a group that has won a war is exactly the same under the two scenarios, but the selective pressure on belligerence and bravery depends on the four quantities discussed above. It is thus a priori not clear which scenario is more conducive to belligerence and bravery, and by how much the selection pressure on the traits will differ between them.

Under the life-cycle assumptions used in this paper, the main quantitative difference in the selective pressure on belligerence and bravery under the two scenarios stems from the fitness benefits of defeating other groups and relatedness taking different values. Under the DGR scenario, the fitness benefits of defeating another group; namely, the increase in group reproductive value, is equal to 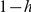, but this value is lower under the VGE and equal to 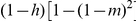 when the benefit of warfare are small. Hence, the benefit of defeating a group decreases as migration decreases under the VGE model. The main reason for this difference is that when the benefits of warfare increase local carrying capacity and there is small migration, a local increase in group size does not contribute much to the far distant future growth rate of the population because the benefits of warfare are not exported but increase competition locally, which reduces group reproductive value (eq. B-29 of Appendix S2). That local competition between individuals within groups can partially or even completely inhibit the benefits obtained by expressing social behaviors is a classical result [29], [42], [56], [58], [59]. By contrast, under the DGR scenario, groups contribute more to the far distant future growth rate of the population by repopulating other groups because this is a form of exportation of the demographics benefits of belligerence and bravery, which are not destroyed locally through an increase in competition. Another way to interpret this is to say that competition is more of the “hard” type under the DGR scenario than under the VGE scenario [60], [61], and thus more conducive to selection on costly social behaviors.

The relatedness coefficient tends to be smaller under the DGR scenario than under the VGE scenario, which now tips the balance in favor of stronger selection on belligerence and bravery under the VGE scenario. This follows from the fact that genetic mixing occurs during repopulation, which decreases the probability of identity within groups, unless groups are completely repopulated (Fig. 2). By contrast, no such mixing occurs under the VGE scenario, where groups remain the same unit after warfare, although an increase in groups size due to gaining more resources also tends to decrease the relatedness within groups because coalescence within groups (probability that two individuals descend from the parent) decreases (Fig. 2). A consequence is that the difference in the relatedness between the two scenarios is not so strong (Fig. 2).

Combining the effect of relatedness and the fitness benefits of defeating other groups on the selective pressure suggests that selection is stronger on both traits under the DGR than under the VGE scenario. From an evolutionary perspective, this suggests that the possibility to repopulate defeated groups by fission of a victorious one or by its members fertilizing the females of defeated groups leads to higher fitness benefits than the gain of additional resources by conquest, which increase local carrying capacity.

The two demographic scenarios of warfare analyzed here may also occur under different ecological conditions. The DGR scenario may be more consistent with a situation where groups live on or around resources that are concentrated and have static positions in space and time (such as patchily distributed resources, water points, or shelters) and fight over them for monopolization. This may be the case in social insects [1] or in hominids living in arid or semiarid environments, where population density is low and water holes were often the main cause of competition [12], [23]. The DGR scenario may also be consistent with situations where bands of males aimed at gaining additional mates by raiding and mating with female from other groups. By contrast, the VGE scenario is more likely to represent a situation where groups fight for animal resources, which were important for meat, clothing, and raw materials used in tool-making [12], [23]. Here, the gained resources can be shared among combatants and put in use in the focal group.

As a final point, the DGR and VGE scenarios are mainly relevant for understanding the fitness benefits of warfare in pre-state societies, where interactions occur between individuals that are more likely to share a recent common ancestor than are individuals sampled at random from the population. But the role of and motivations behind belligerence and bravery in the transition from small-scale to large-scale societies remains to be better understood. In the absence of benefits to self or relatives, there should be no naturally selected motivations in the world causing an individual to lay down its life.

## Supporting Information

Appendix S1 Derivation of the fighting probabilities.
(PDF)

Appendix S2 Derivation of the selective pressures on belligerence and bravery under the VGE demographic scenario.
(PDF)
