# Supplementary material for: The Demographic Benefits of Belligerence and Bravery: Defeated Group Repopulation or Victorious Group Size Expansion?
Source: PLoS One. 2011 Jul 5;6(7):e21437. doi: 10.1371/journal.pone.0021437 (PMC3130041; doi:10.1371/journal.pone.0021437)
Supplement: Appendix S1 — Derivation of the fighting probabilities. (PDF) [file pone.0021437.s001.pdf]

Appendix S1: from attack rates to pairwise fighting probabilities

In this appendix, I present the derivation of the probability that a focal group engages in a fight with another one under the assumption that each group attacks another random group from the population independently of each other. Further, during a single generation each group is assumed to engage at most into a single pairwise contest when it is in the role of a defender (see section "Fighting probability" of the main text); there is thus only a one-shot local contest when a given group is attacked.

I first consider the case where the total population consists of a finite number n_{d} of groups and then let the number of groups grow very large ($n_{\mathrm{d}} \to \infty$). With the finite number of groups assumption, the probability that a focal group is not attacked when the attack rate is a for each group in the population is $[1 - a/(n_{\mathrm{d}} - 1)]^{n_{\mathrm{d}}-1}$, where $a/(n_{\mathrm{d}} - 1)$ is the probability that a random group from the population attacks the focal group. In the limit of an infinite number of groups, the probability that the focal group is not attacked is then given by $\exp(-a)$, which is obtained by using the standard relation $\lim_{n\to\infty}(1-x/n)^n \to \exp(-x)$. The probability that a focal group is attacked by another group and that a fight occurs when it is in the role of a defender is then $1 - \exp(-a)$.

With probability $a/(n_{\mathrm{d}}-1)$, the focal group attacks another random group from the population. When this event occurs and k other groups from the population attack the same group as the focal group, the probability that the focal group gets into a fight with the attacked group is given by $1/(1 + k)$, where $1+k$ is the total number of groups (including the focal) attacking the same group. Hence, conditional on the focal group attacking another random group from the population, the probability that it will engage into a contest with the attacked group is given by the average of $1/(1 + k)$ over a Binomial distribution, which gives the number of simultaneous attacks on the group attacked by the focal group. This yields the average

$$q = \sum_{k=0}^{n_{\mathrm{d}}-2} \frac{1}{1+k} \frac{(n_{\mathrm{d}}-2)!}{k!(n_{\mathrm{d}}-2-k)!} \left(\frac{a}{n_{\mathrm{d}}-1}\right)^{k} \left(1-\frac{a}{n_{\mathrm{d}}-1}\right)^{n_{\mathrm{d}}-2-k}, \tag{A-1}$$

where $n_{\mathrm{d}} - 2$ is the number of groups in the population without the focal group and the group it has attacked.

Using the fact that $(n_{\mathrm{d}} - 2)! = (n_{\mathrm{d}} - 1)!/(n_{\mathrm{d}} - 1)$, eq. A-1 can be written as

$$q = \frac{1}{a} \sum_{k=0}^{n_{\mathrm{d}}-2} \frac{(n_{\mathrm{d}}-1)!}{(1+k)![n_{\mathrm{d}}-1-(1+k)]!} \left(\frac{a}{n_{\mathrm{d}}-1}\right)^{1+k} \left(1-\frac{a}{n_{\mathrm{d}}-1}\right)^{n_{\mathrm{d}}-1-(1+k)}. \tag{A-2}$$

I now make the change of variable $h = k + 1$ in the summation sign, which gives

$$q = \frac{1}{a} \sum_{h=1}^{n_{\mathrm{d}}-1} \frac{(n_{\mathrm{d}}-1)!}{h!(n_{\mathrm{d}}-1-h)!} \left(\frac{a}{n_{\mathrm{d}}-1}\right)^{h} \left(1-\frac{a}{n_{\mathrm{d}}-1}\right)^{n_{\mathrm{d}}-1-h}, \tag{A-3}$$

and shows that the sum is over a Binomial distribution with probability of success $a/(n_{\mathrm{d}}-1)$ and number of draws $n_{\mathrm{d}} - 1$. Since the sum runs from $h = 1$ to $h = n_{\mathrm{d}} - 1$, the sum gives the probability that there is at least one success in $n_{\mathrm{d}} - 1$ draws, whereby

$$q = \frac{1}{a}\left[1 - \left(1 - \frac{a}{n_{\mathrm{d}}-1}\right)^{n_{\mathrm{d}}-1}\right]. \tag{A-4}$$

By letting the number of groups grow large ($n_{\mathrm{d}} \to \infty$) and using again $\lim_{n\to\infty}(1 - x/n)^n \to \exp(-x)$ the probability that a focal group, conditional on it attacking another group, enters into a contest with the attacked group is given by $[1 - \exp(-a)]/a$. Since the focal group can attack $n_{\mathrm{d}} - 1$ groups, the probability that the focal groups enters into a fight when it is in the role of an attacker in a monorophic population is $(n_{\mathrm{d}} - 1) \times a/(n_{\mathrm{d}} - 1) \times [1 - \exp(-a)]/a = 1 - \exp(-a)$.

If we now call $\phi(i,j)$ the probability that a focal group with average level of belligerence i, and thus attack rate $a(i)$, enters into a fight with another group when j is the average level of belligerence in the remaining groups in the population, which thus have attack rate $a(j)$, then the above results give

$$\phi(i,j) = a(i)\frac{1-\exp(-a(j))}{a(j)}. \tag{A-5}$$
