# Supplementary material for: The Demographic Benefits of Belligerence and Bravery: Defeated Group Repopulation or Victorious Group Size Expansion?
Source: PLoS One. 2011 Jul 5;6(7):e21437. doi: 10.1371/journal.pone.0021437 (PMC3130041; doi:10.1371/journal.pone.0021437)
Supplement: Appendix S2 — Derivation of the selective pressures on belligerence and bravery under the VGE demographic scenario. (PDF) [file pone.0021437.s002.pdf]

Appendix S2: VGE model

Selection gradient

In this appendix, I present the derivation of the selective pressures on belligerence and bravery under the VGE demographic scenario. For this scenario, the demographic events s_1, s_2, s_3, and s_4, determine the number of individuals that reach adulthood in a group after stage 4 of the life cycle. For the events s_1, s_2, s_3, and s_4, the group size that obtains is, respectively, $N + (1-h)N, N, N$, and hN (see section "Demographic events" of the main text). A focal group is thus likely to fluctuate between different sizes across generations.

These demographic fluctuations make the calculations of the selection gradient on an evolving phenotype z more complicated. In the presence of such demographic fluctuations and if the number of juveniles produced by each individual is assumed to be large (or is Poisson distributed), the selection gradient can be expressed as

$$S = S_{\mathrm{f}} + S_{\mathrm{Pr}} \tag{B-1}$$

[1], where

$$S_{\mathrm{f}} = \sum_{s}\sum_{s'} \nu(s') \left[\frac{\partial f_{\mathrm{p}}(s)}{\partial z_{\bullet}} + \frac{\partial f_{\mathrm{p}}(s)}{\partial z_0^{\mathrm{R}}} R^{\mathrm{R}}(s) + \sum_{s^*} \Pr(s^*) \left(\frac{\partial f_{\mathrm{d}}(s,s^*)}{\partial z_{\bullet}} + \frac{\partial f_{\mathrm{d}}(s,s^*)}{\partial z_0^{\mathrm{R}}} R^{\mathrm{R}}(s^*) \right) \right] \Pr(s' \mid s) \Pr(s) \tag{B-2}$$

and

$$S_{\mathrm{Pr}} = \sum_{s}\sum_{s'} \nu(s') \frac{\partial \Pr(s' \mid s)}{\partial z_0^{\mathrm{R}}} f_{\mathrm{p}}(s) R^{\mathrm{R}}(s) \Pr(s), \tag{B-3}$$

where all sums are over all elements s of the set $\mathcal{S} = \{s_1, s_2, s_3, s_4\}$ of demographic events (see Fig. 1), and where the components of selection follow from rearranging eqs. 26–27 of [1] as given by eqs. A-48–A-49 of [2]. All partial derivatives in eqs. B-2–B-3 are evaluated at the phenotypic value of the resident allele ($z_{\bullet} = z_0^{\mathrm{R}} = z_1 = z$) and all other quantities in S are also evaluated at this neutrality point.

Because the four demographic events determine the state of a focal group (its size), I will from now on, and for ease of presentation, refer to the four events $s1$, $s2$, $s3$, and $s4$, as the demographic states of a group. The size of a focal group is the main variable that will affect the components of selection given by eq. B-2 and eq. B-3, and which depend on four type of quantities, the justification of which are given in [1].

(1) Two frequency functions. First, the probability $f_{\mathrm{p}}(s)$ that a gene sampled in a group, which is in a given state s' in the offspring generation, descends from an individual from that group that was in state s in the parental generation. This probability is independent of the states of the group in the offspring generation [1], which follows from the assumption that fecundity is Poisson distributed (or very large). The second frequency function is the probability $f_{\mathrm{d}}(s, s^*)$ that a gene sampled in a group, which is in a given state s' in the offspring generation and was itself in state s in the parental generation, descends through migration from a group in state s^* in the parental generation. For completeness, I mention that these frequency functions can be related to individual fitness functions, which measure the expected number of offspring reaching adulthood of a focal parent conditional on the same demographic events described for the frequency functions, by $w_{\mathrm{p}}(s, s') = f_{\mathrm{p}}(s)N_{s'}/N_s$, $w_{\mathrm{d}}(s, s', s^*) = f_{\mathrm{d}}(s, s^*)N_{s'}/N_{s^*}$ [1], where N_s is the number of individuals in a group in demographic state s.

(2) The stationary probability $\Pr(s)$ that a focal group of individuals is in demographic state s, which is the steady-state distribution of the Markov chain with forward transitions probabilities $\Pr(s' \mid s)$, where

s is the demographic state of the group in the parental generation and s' is its state in the offspring generation. This stationary distribution satisfies

$$\Pr(s') = \sum_{s} \Pr(s' \mid s) \Pr(s). \tag{B-4}$$

(3) The relative reproductive value $\nu(s)$ of all individuals within a group in demographic state s, which satisfies the recursion

$$\nu(s) = \sum_{s'} \left[f_{\mathrm{p}}(s) \Pr(s' \mid s) + \sum_{s^*} f_{\mathrm{d}}(s^*, s) \Pr(s' \mid s^*) \Pr(s^*) \right] \nu(s') \tag{B-5}$$

and $\sum_s \nu(s) \Pr(s) = 1$, where $\nu(s) \Pr(s)$ is the reproductive value of all groups in state s in the population. In the special case where the demographic states are independently distributed across generations $\Pr(s' \mid s^*) = \Pr(s')$, in which case eq. B-5 can be simplified by using $\nu(s) \Pr(s)$ so that the reproductive value of a group in state s is directly given by

$$\nu(s) = f_{\mathrm{p}}(s) + \sum_{s^*} f_{\mathrm{d}}(s^*, s) \Pr(s^*). \tag{B-6}$$

(4) The probability of identity by descent $R^{\mathrm{R}}(s)$ between two homologous genes sampled with replacement in a group in state s. This probability can be expressed as

$$R^{\mathrm{R}}(s) = \frac{1}{N_s} + \left(\frac{N_s - 1}{N_s} \right) R(s), \tag{B-7}$$

where N_s is the number of individuals in a group in demographic state s and $R(s)$ is the probability of identity between two homologous genes sampled without replacement in that group. This probability satisfies the recursion

$$R(s') = \sum_{s} \Pr(s \mid s') f_{\mathrm{p}}(s)^2 R^{\mathrm{R}}(s), \tag{B-8}$$

where $\Pr(s \mid s')$ is the probability that a group in state s' in the offspring generation derives from a group in state s in the parental generation [backward transition probability of the demographic states $\Pr(s \mid s') = \Pr(s' \mid s) \Pr(s) / \Pr(s')$], and $f_{\mathrm{p}}(s)^2$ is the backward migration probability that a pair of genes sampled in a group in a given state s' in the offspring generation both descend from the same group that was in state s in the parental generation.

Frequency functions

For the life-cycle assumptions described in the main text, the frequency functions are given by

$$f_{\mathrm{p}}(s) = \frac{(1-m) b_{\bullet} N_s}{(1-m)\, b_{\mathrm{r}} N_s + m b_{\mathrm{d}} N_{\mathrm{eq}}} \tag{B-9}$$

and

$$f_{\mathrm{d}}(s, s^*) = \frac{m b_{\bullet} N_{s^*}}{(1-m)\, b_{\mathrm{d}} N_s + m b_{\mathrm{d}} N_{\mathrm{eq}}}, \tag{B-10}$$

where $N_{\mathrm{eq}} = \sum_s N_s \Pr(s)$ is the average group size in the population [1, eqs. 33–34] with the number of adults in the different demographics states being given by $N_{s_1} = [1 + (1-h)]\, N$, $N_{s_3} = [h + (1-h)]\, N = N$, $N_{s_2} = N$, and $N_{s_4} = hN$.

On substitution of these numbers into eq. B-9 one has

$$
\begin{aligned}
f_{\mathrm{p}}(s_1) &= \frac{(1-m)b_{\bullet}[1+(1-h)]}{(1-m)\,b_{\mathrm{r}}[1+(1-h)]+m\,b_{\mathrm{d}}\,(N_{\mathrm{eq}}/N)} \\
f_{\mathrm{p}}(s_3) &= \frac{(1-m)b_{\bullet}}{(1-m)\,b_{\mathrm{r}}+m\,b_{\mathrm{d}}\,(N_{\mathrm{eq}}/N)} \\
f_{\mathrm{p}}(s_2) &= \frac{(1-m)b_{\bullet}}{(1-m)\,b_{\mathrm{r}}+m\,b_{\mathrm{d}}\,(N_{\mathrm{eq}}/N)} \\
f_{\mathrm{p}}(s_4) &= \frac{(1-m)b_{\bullet}h}{(1-m)\,b_{\mathrm{r}}h+m\,b_{\mathrm{d}}\,(N_{\mathrm{eq}}/N)},
\end{aligned} \tag{B-11}
$$

and similar calculations lead to explicit expressions for the frequency functions for the migrant offspring $[f_{\mathrm{d}}(\cdot,\cdot)]$.

Assuming a population of resident individuals and substituting the frequency functions into eq. B-5 results in complicated expressions but they can be handled with a symbolic algebra system like Mathematica [3]. Assuming that the demographic states are independently distributed across generations, eq. B-6 still results in complicated expressions but when dispersal is complete ($m = 1$), the group reproductive values reduce to

$$\nu(s_1) = 1 + (1-h), \nu(s_2) = 1, \nu(s_3) = 1, \text{ and } \nu(s_4) = h, \tag{B-12}$$

which is similar to those obtained under the DGR scenario (eq. 13 of the main text).

Transition probabilities

In order to evaluate S, we also need the transition probabilities of the demographic states for a focal group, which, as for the DGR scenario, will depend on the fighting and winning probabilities. The fighting probabilities between groups are assumed to be the same as those in the DGR scenario (eq. 1 of the main text or eq. A-5 of Appendix S1), with the only difference that the average phenotype among subadults in the focal group after dispersal may now depend on the demographic states because the frequency of individuals in the focal group after dispersal and descending from the focal group may depend on the size of that group in the parental generation.

When warfare occurs before the dispersal of subadults, we have $x_0^{\mathrm{A}} = x_0^{\mathrm{R}}$ and $y_0^{\mathrm{A}} = y_0^{\mathrm{R}}$, but when warfare occurs after the dispersal of subadults, we have to take into account that migration has changed the average phenotype in the focal group (see eq. 2 of the main text). The average level of belligerence in the focal group when the size of the focal group in the parental generation is N_s is $x_0^{\mathrm{A}}(s) = [(1-m)N_s]/[(1-m)\,N_s + mN_{\mathrm{eq}}]x_0^{\mathrm{R}} + [mN_{\mathrm{eq}}]/[(1-m)\,N_s + mN_{\mathrm{eq}}]x_1$, where $[(1-m)N_s]/[(1-m)\,N_s + mN_{\mathrm{eq}}]$ is the probability of sampling an individual of philopatric origin in the focal group after dispersal. Note that x_1 does not depend on demographic states because any class of individuals from other groups have zero relatedness to the focal individual so that their average phenotypes can be considered to be equivalent when viewed from the focal group and taken to be equal to x_1. In order to take simultaneously into account the case where warfare occurs before and after the dispersal of subadults (e.g. eq. 3 of the main text), I write the average phenotypes of subadults in the focal group as

$$
\begin{aligned}
x_0^{\mathrm{A}}(s) &= \frac{(1-m\epsilon)N_s}{(1-m\epsilon)\,N_s + m\epsilon N_{\mathrm{eq}}}x_0^{\mathrm{R}} + \frac{m\epsilon N_{\mathrm{eq}}}{(1-m\epsilon)\,N_s + m\epsilon N_{\mathrm{eq}}}x_1 \\
y_0^{\mathrm{A}}(s) &= \frac{(1-m\epsilon)N_s}{(1-m\epsilon)\,N_s + m\epsilon N_{\mathrm{eq}}}y_0^{\mathrm{R}} + \frac{m\epsilon N_{\mathrm{eq}}}{(1-m\epsilon)\,N_s + m\epsilon N_{\mathrm{eq}}}y_1,
\end{aligned} \tag{B-13}
$$

where the parameter ϵ is set to zero when warfare occurs before the dispersal of offspring and set to one if it occurs after the dispersal of offspring.

With probability $\phi_0(s) \equiv \phi(x_0^{\mathrm{A}}(s), x_1)$ the subadults in a focal group, which was in demographic state s in the parental generation, enter into a fight with another group by attacking. The focal group is assumed to win the ensuing battle with probability

$$v_0(s) = \sum_{s^*} \Pr(s^*) \frac{\omega g\big(y_0^{\mathrm{A}}(s) N_s\big)}{\omega g\big(y_0^{\mathrm{A}}(s) N_s\big) + (1-\omega) g(y_1 N_{s^*})}, \tag{B-14}$$

which is an average over all possible contests faced by the individuals in the focal group at steady-state. This equation is the direct extension of eq. 5 of the main text to the case where opposing groups vary in size, and is then averaged over the distribution $\Pr(s^*)$ of demographic states of the attacked group evaluated in the neutral process. Taking such an average can be justified by the fact that owing to the infinite island model assumptions, groups affect each other in a nonstochastic manner, only through limiting densities, which can be taken as those of the focal deme itself [1, 4].

With probability $\phi_1 \equiv \phi(x_1, x_1)$ a focal group in demographic state s is attacked by another group from the population, in which case the probability that the attacking group wins the ensuing battle is

$$v_1(s) = \sum_{s^*} \Pr(s^*) \frac{\omega g(y_1 N_{s^*})}{(1-\omega) g\big(y_0^{\mathrm{A}}(s) N_s\big) + \omega g(y_1 N_{s^*})}, \tag{B-15}$$

which is an average over all demographic states of the attacker group.

With these notations, the forward transition probabilities, from a focal group in demographic state s in the parental generation to any of the four other states in the offspring generation are given by

$$\begin{aligned}
\Pr(s_1 \mid s) &= [1 - \phi_1 v_1(s)]\, \phi_0(s) v_0(s) \\
\Pr(s_2 \mid s) &= \phi_1 v_1(s) \phi_0(s) v_0(s) \\
\Pr(s_3 \mid s) &= [1 - \phi_1 v_1(s)][1 - \phi_0(s) v_0(s)] \\
\Pr(s_4 \mid s) &= \phi_1 v_1(s)[1 - \phi_0(s) v_0(s)].
\end{aligned} \tag{B-16}$$

The stationary distribution (eq. B-4) induced by this Markov chain is difficult to analyze because the transition probabilities themselves depend on the stationary distribution (e.g., eq. B-14) so that the stationary distribution it is only implicitly determined and cannot be evaluated by using linear algebra. This is a non-homogeneous Markov chain that is homogeneous only asymptotically.

Selection: effect on settled offspring number S_{f}

The frequency functions (eq. B-9 and eq. B-10) describe a model of local group size fluctuations with Wright-Fisher reproduction [1,2]. For this case, it has been shown that the first component of the selection gradient on a phenotype z, S_{f}, can be simplified and expressed solely in terms of the perturbations of the relative fecundity of a focal individual (number of juveniles produced before a competition stage) as

$$S_{\mathrm{f}} = \sum_s \frac{1}{b} \left(\frac{\partial b_\bullet}{\partial z_\bullet} + \frac{\partial b_\bullet}{\partial z_0^{\mathrm{R}}} \frac{1}{N_s} \right) \nu(s) \left(1 - R(s)\right) \Pr(s) \tag{B-17}$$

[2, eq. 21 of the Appendix].

In the present model, we further have $\partial b_\bullet / \partial z_0^{\mathrm{R}} = 0$, whereby

$$S_{\mathrm{f}} = \frac{1}{b} \frac{\partial b_\bullet}{\partial z_\bullet} \sum_s \nu(s) \left(1 - R(s)\right) \Pr(s), \tag{B-18}$$

which can be used to evaluate both the selection gradient on belligerence and bravery by using $b_\bullet = [1 - C_{\mathrm{x}}(x_\bullet)]\,[1 - C_{\mathrm{y}}(y_\bullet)]$.

Selection: effect on offspring reproductive value S_{Pr}

Belligerence

Using eq. B-13 to describe the phenotype of subadult individuals and holding bravery constant ($y_0^{\mathrm{R}} = y_1 = y$), the perturbations of the transition probabilities (eq. B-16) due to expressing belligerence and evaluated at the neutrality point ($x_0^{\mathrm{R}} = x_1 = x$) are given by

$$\begin{aligned}
\frac{\Pr(s_1 \mid s)}{\partial x_0^{\mathrm{R}}} &= \frac{\partial \phi_0(s)}{\partial x_0^{\mathrm{R}}} v_0(s) \left[(1 - \phi v_1(s)\right] \\
\frac{\Pr(s_2 \mid s)}{\partial x_0^{\mathrm{R}}} &= \frac{\partial \phi_0(s)}{\partial x_0^{\mathrm{R}}} v_0(s) \phi v_1(s) \\
\frac{\Pr(s_3 \mid s)}{\partial x_0^{\mathrm{R}}} &= -\frac{\Pr(s_1 \mid s)}{\partial x_0^{\mathrm{R}}} \\
\frac{\Pr(s_4 \mid s)}{\partial x_0^{\mathrm{R}}} &= -\frac{\Pr(s_2 \mid s)}{\partial x_0^{\mathrm{R}}}.
\end{aligned} \tag{B-19}$$

Substituting these expressions into eq. B-3 and rearranging produces

$$\begin{aligned}
S_{\mathrm{Pr}} = &\sum_s f_{\mathrm{p}}(s) R^{\mathrm{R}}(s) \Pr(s) \\
&\times \frac{\partial \phi_0(s)}{\partial x_0^{\mathrm{R}}} v_0(s) \Big[\{1 - \phi v_1(s)\}\{\nu(s_1) - \nu(s_3)\} + \phi v_1(s)\{\nu(s_2) - \nu(s_4)\}\Big].
\end{aligned} \tag{B-20}$$

Bravery

Using eq. B-13 in eq. B-16 and holding belligerence constant, the perturbations of the transition probabilities evaluated at the neutrality point can be written as

$$\begin{aligned}
\frac{\Pr(s_1 \mid s)}{\partial y_0^{\mathrm{R}}} &= \phi \left[\{1 - \phi v_1(s)\} \frac{\partial v_0(s)}{\partial y_0^{\mathrm{R}}} - \phi v_0(s) \frac{\partial v_1(s)}{\partial y_0^{\mathrm{R}}}\right] \\
\frac{\Pr(s_2 \mid s)}{\partial y_0^{\mathrm{R}}} &= \phi^2 \left[v_1(s) \frac{\partial v_0(s)}{\partial y_0^{\mathrm{R}}} + v_0(s) \frac{\partial v_1(s)}{\partial y_0^{\mathrm{R}}}\right] \\
\frac{\Pr(s_3 \mid s)}{\partial y_0^{\mathrm{R}}} &= -\frac{\Pr(s_1 \mid s)}{\partial y_0^{\mathrm{R}}} - \phi \frac{\partial v_1(s)}{\partial y_0^{\mathrm{R}}} \\
\frac{\Pr(s_4 \mid s)}{\partial y_0^{\mathrm{R}}} &= -\frac{\Pr(s_2 \mid s)}{\partial y_0^{\mathrm{R}}} + \phi \frac{\partial v_1(s)}{\partial y_0^{\mathrm{R}}}.
\end{aligned} \tag{B-21}$$

Substituting these expressions into eq. B-3 and rearranging produces

$$\begin{aligned}
S_{\mathrm{Pr}} = &\sum_s f_{\mathrm{p}}(s) R^{\mathrm{R}}(s) \Pr(s) \Big[\{\nu(s_1) - \nu(s_3)\} \frac{\Pr(s_1 \mid s)}{\partial y_0^{\mathrm{R}}} \\
&+ \{\nu(s_2) - \nu(s_4)\} \frac{\Pr(s_2 \mid s)}{\partial y_0^{\mathrm{R}}} + \{\nu(s_4) - \nu(s_3)\} \phi \frac{\partial v_1(s)}{\partial y_0^{\mathrm{R}}}\Big].
\end{aligned} \tag{B-22}$$

Approximate functionals

Stationary distribution

By using eq. B-16, I was unable to obtain analytical expression for the stationary demographic distribution $\Pr(s)$ under neutrality, which we need in order to evaluate eq. B-2 and eq. B-3. Without an analytical

expression of the stationary distribution there is no hope to evaluate explicitly the selection gradients on belligerence and bravery.

However, numerical exploration of the stationary distribution suggests that it is weakly affected by the parameter h when $h > 0.5$ and that the probabilities of occurrences of the demographic states take values very close to that if h were equal to one in that case. An approximation for the Markov chain described by eq. B-16 for large h values can then be obtained by carrying out a first order Taylor expansion around $h = 1$ of the transition probabilities given by eq. B-16, which was done with Mathematica [3]. Substituting the resulting transition probabilities into eq. B-4 and solving for the stationary distribution gives

$$\begin{aligned}
\Pr(s_1) &= (1-\phi\omega)\,\phi\omega + O\left((1-h)^2\right) \\
\Pr(s_2) &= \phi^2\omega^2 + O\left((1-h)^2\right) \\
\Pr(s_3) &= (1-\phi\omega)^2 + O\left((1-h)^2\right) \\
\Pr(s_4) &= \phi\omega(1-\phi\omega) + O\left((1-h)^2\right),
\end{aligned} \tag{B-23}$$

where $O\left((1-h)^2\right)$ is a remainder of order $(1-h)^2$. In the rest of this Appendix, I evaluate the components of the selection gradients on belligerence and bravery to the first order in $1-h$ so that eq. B-23 can be used in order to evaluate eq. B-2 and eq. B-3 to the first order in h around $h = 1$.

Winning and fighting probabilities

A first order Taylor expansion of the fighting probability around $h = 1$ gives

$$\begin{aligned}
\frac{\partial \phi_0(s)}{\partial x_0^{\mathrm{R}}} &= \frac{\partial \phi_0(s)}{\partial x_0^{\mathrm{A}}(s)} \frac{\partial x_0^{\mathrm{A}}(s)}{\partial x_0^{\mathrm{R}}} \\
&= (1-\epsilon m)\phi' + O\left(1-h\right),
\end{aligned} \tag{B-24}$$

where $\phi' \equiv \partial \phi_0(s)/\partial x_0^{\mathrm{A}}(s)$.

Substituting eq. B-23 into eqs. B-14–B-15 gives

$$\begin{aligned}
\frac{\partial v_0(s)}{\partial y_0^{\mathrm{R}}} &= \frac{\partial v_0(s)}{\partial y_0^{\mathrm{A}}(s)} \frac{\partial y_0^{\mathrm{A}}(s)}{\partial y_0^{\mathrm{R}}} \\
&= (1-\epsilon m)v' + O\left(1-h\right),
\end{aligned} \tag{B-25}$$

where $v' \equiv N\omega(1-\omega)g'/g$, and

$$\begin{aligned}
\frac{\partial v_1(s)}{\partial y_0^{\mathrm{R}}} &= \frac{\partial v_1(s)}{\partial y_0^{\mathrm{A}}(s)} \frac{\partial y_0^{\mathrm{A}}(s)}{\partial y_0^{\mathrm{R}}} \\
&= -(1-\epsilon m)v' + O\left(1-h\right).
\end{aligned} \tag{B-26}$$

In a monormorphic population we also have

$$\begin{aligned}
v_0(s) &= \omega + O\left(1-h\right) \\
v_1(s) &= \omega + O\left(1-h\right).
\end{aligned} \tag{B-27}$$

With these results, eq. B-21 reduces to

$$\begin{aligned}
\frac{\Pr(s_1 \mid s)}{\partial y_0^{\mathrm{R}}} &= \phi v'(1-\epsilon m) + O\left(1-h\right) \\
\frac{\Pr(s_2 \mid s)}{\partial y_0^{\mathrm{R}}} &= O\left(1-h\right) \\
\frac{\Pr(s_3 \mid s)}{\partial y_0^{\mathrm{R}}} &= O\left(1-h\right) \\
\frac{\Pr(s_4 \mid s)}{\partial y_0^{\mathrm{R}}} &= -\phi v'(1-\epsilon m) + O\left(1-h\right). \qquad \text{(B-28)}
\end{aligned}$$

Reproductive values

Substituting eqs. B-9–B-10, eq. B-16, and eq. B-23 into eq. B-5 and solving for the reproductive values provides the following first order Taylor expansion around $h = 1$ at neutrality:

$$\begin{aligned}
\nu(s_1) &= 1 + (1-h)\left(1-(1-m)^2\right) + O\Big((1-h)^2\Big) \\
\nu(s_2) &= 1 + O\Big((1-h)^2\Big) \\
\nu(s_3) &= 1 + O\Big((1-h)^2\Big) \\
\nu(s_4) &= 1 - (1-h)\left(1-(1-m)^2\right) + O\Big((1-h)^2\Big), \qquad \text{(B-29)}
\end{aligned}$$

which gives

$$\begin{aligned}
\nu(s_1) - \nu(s_3) &= (1-h)\left(1-(1-m)^2\right) + O\Big((1-h)^2\Big) \\
\nu(s_2) - \nu(s_4) &= (1-h)\left(1-(1-m)^2\right) + O\Big((1-h)^2\Big) \\
\nu(s_4) - \nu(s_3) &= -(1-h)\left(1-(1-m)^2\right) + O\Big((1-h)^2\Big). \qquad \text{(B-30)}
\end{aligned}$$

These expressions are needed to evaluate the selection pressure S_{Pr} for both belligerence and bravery (eq. B-20 and eq. B-22). Eq. B-30 illustrates that the changes in reproductive value involve terms of order $(1-h)$. Hence, in order to evaluate S_{Pr} to the first order around $h = 1$, it is sufficient to evaluate all other functionals ($f_{\mathrm{p}}(s)$, $R^{\mathrm{R}}(s)$, $\Pr(s)$, etc.) to the zero's order as any term of order $(1-h)$ in these functionals would result in second order terms in the selection gradient.

Probabilities of identity by descent

Substituting eq. B-10, eq. B-16, and eq. B-23 into eq. B-8 and solving for relatedness provides the following first order Taylor expansions

$$R(s) = \frac{(1-m)^2}{N-(N-1)(1-m)^2} + O\Big((1-h)^2\Big) \qquad \text{(B-31)}$$

and

$$\begin{aligned}
R_{\mathrm{p}} &\equiv \sum_s f_{\mathrm{p}}(s) R^{\mathrm{R}}(s) \Pr(s) \\
&= \frac{(1-m)}{N-(N-1)(1-m)^2} + O\Big((1-h)^2\Big). \qquad \text{(B-32)}
\end{aligned}$$

Approximate selection gradient

We now have all the elements to evaluate the selection gradients on belligerence and bravery to the first order around $h = 1$.

Selection on belligerence

Holding bravery constant and using belligerence as the evolving trait, eq. B-18 reduces to

$$S_{\mathrm{f}} = -\frac{C_{\mathrm{x}}'}{1 - C_{\mathrm{x}}}(1 - R) + O\Big((1-h)^2\Big), \tag{B-33}$$

because $\sum_s \nu(s)\,\Pr(s) = 1$ and $R(s)$ is the same for all s (see eq. B-31) and is denoted R.

Substituting eq. B-30, eq. B-24, and eq. B-27 into eq. B-20 gives

$$S_{\mathrm{Pr}} = \sum_s f_{\mathrm{p}}(s) R^{\mathrm{R}}(s)\Pr(s) \times (1-\epsilon m)\phi'\omega\Big[(1-\phi\omega)(1-h)\left(1-(1-m)^2\right) + \phi\omega(1-h)\left(1-(1-m)^2\right)\Big], \tag{B-34}$$

which can be simplified to

$$S_{\mathrm{Pr}} = \phi'\omega(1-h)\left(1-(1-m)^2\right)(1-\epsilon m)R_{\mathrm{p}} + O\Big((1-h)^2\Big) \tag{B-35}$$

by using eq. B-32. The coefficient R_{p} is the average probability of identity between a gene sampled in a philopatric individual (individual that has not dispersed) and a homologous gene sampled in a neighbour.

Adding up S_{f} and S_{Pr}, and taking into account only first order terms, one obtains the inclusive fitness effect from expressing belligerence as

$$S = -\frac{C_{\mathrm{x}}'}{1 - C_{\mathrm{x}}}(1 - R) + \phi'\omega(1-h)\left(1-(1-m)^2\right)(1-\epsilon m)R_{\mathrm{p}}. \tag{B-36}$$

Setting the selective pressure to zero and neglecting terms of order $(1-h)^2$ and of higher order, the cost-to-benefit ratio under which belligerence spreads can be written as

$$\frac{C_{\mathrm{x}}'}{1 - C_{\mathrm{x}}} = \frac{\phi'\omega(1-h)\left(1-(1-m)^2\right)(1-\epsilon m)R_{\mathrm{p}}}{1-R}, \tag{B-37}$$

where using eqs. B-31–B-32 produces

$$\frac{R_{\mathrm{p}}}{1-R} = \frac{(1-m)}{\left(1-(1-m)^2\right)N}. \tag{B-38}$$

Selection on bravery

Following similar calculations as in the last section, we have from eq. B-18 for bravery that

$$S_{\mathrm{f}} = -\frac{C_{\mathrm{y}}'}{1 - C_{\mathrm{y}}}(1 - R). \tag{B-39}$$

Using eq. B-30 and eqs. B-26–B-28 in eq. B-20, and holding belligerence constant produces

$$S_{\mathrm{Pr}} = \sum_s f_{\mathrm{p}}(s) R^{\mathrm{R}}(s)\Pr(s)\Big[(1-h)\left(1-(1-m)^2\right)\phi(1-\epsilon m)v' + (1-h)\left(1-(1-m)^2\right)\phi(1-\epsilon m)v'\Big] + O\Big((1-h)^2\Big), \tag{B-40}$$

whereby

$$S_{\mathrm{Pr}} = \phi v' 2(1-h)\left(1-(1-m)^2\right)(1-\epsilon m)R_{\mathrm{p}} + O\Big((1-h)^2\Big). \tag{B-41}$$

Adding up the selective pressures one obtains

$$S = -\frac{C'_{\mathrm{y}}}{1-C_{\mathrm{y}}}(1-R) + v' 2(1-h)\left(1-(1-m)^2\right)(1-\epsilon m)R_{\mathrm{p}} + O\Big((1-h)^2\Big). \tag{B-42}$$

Using eq. B-38, eq. B-39, and eq. B-42 in this selection gradient, neglecting the remainder, and setting $S = 0$ finally gives

$$\frac{C'_{\mathrm{y}}}{1-C_{\mathrm{y}}} = \frac{\phi v' 2(1-h)(1-\epsilon m)(1-m)}{N}. \tag{B-43}$$

References

1. Rousset F, Ronce O (2004) Inclusive fitness for traits affecting metapopulation demography. Theoretical Population Biology 65: 127-141.

2. Lehmann L, Perrin N, Rousset F (2006) Population demography and the evolution of helping behaviors. Evolution 60: 1137-1151.

3. Wolfram S (2003) Mathematica, 5th ed. Cambridge: Cambridge University Press.

4. Chesson PL (1981) Models for spatially distributed populations: the effect of within-patch variability. Theoretical Population Biology 19: 288-325.
